# Molecular Identification, Genotypic Diversity, Antifungal Susceptibility, and Clinical Outcomes of Infections Caused by Clinically Underrated Yeasts, *Candida orthopsilosis*, and *Candida metapsilosis*: An Iranian Multicenter Study (2014–2019)

**DOI:** 10.3389/fcimb.2019.00264

**Published:** 2019-07-30

**Authors:** Amir Arastehfar, Sadegh Khodavaisy, Farnaz Daneshnia, Mohammad-Javad Najafzadeh, Shahram Mahmoudi, Arezoo Charsizadeh, Mohammad-Reza Salehi, Hossein Zarrinfar, Abbas Raeisabadi, Somayeh Dolatabadi, Zahra Zare Shahrabadi, Kamiar Zomorodian, Weihua Pan, Ferry Hagen, Teun Boekhout

**Affiliations:** ^1^Department of Medical Mycology, Westerdijk Fungal Biodiversity Institute, Utrecht, Netherlands; ^2^Zoonoses Research Center, Research Institute for Health Development, Kurdistan University of Medical Sciences, Sanandaj, Iran; ^3^Department of Medical Parasitology and Mycology, School of Public Health, Tehran University of Medical Sciences, Tehran, Iran; ^4^Department of Medical Mycology and Parasitology, School of Medicine, Mashhad University of Medical Sciences, Mashhad, Iran; ^5^Students' Scientific Research Center, Tehran University of Medical Sciences, Tehran, Iran; ^6^Immunology, Asthma, and Allergy Research Institute, Tehran University of Medical Sciences, Tehran, Iran; ^7^Department of Infectious Diseases and Tropical Medicine, Faculty of Medicine, Tehran University of Medical Sciences, Tehran, Iran; ^8^Allergy Research Center, Mashhad University of Medical Sciences, Mashhad, Iran; ^9^Faculty of Engineering, Sabzevar University of New Technology, Sabzevar, Iran; ^10^Basic Sciences in Infectious Diseases Research Center, Shiraz University of Medical Sciences, Shiraz, Iran; ^11^Medical Mycology, Shanghai Changzheng Hospital, Second Military Medical University, Shanghai, China; ^12^Department of Medical Microbiology, University Medical Center Utrecht, Utrecht, Netherlands; ^13^Laboratory of Medical Mycology, Jining No. 1 People's Hospital, Jining, China; ^14^Institute of Biodiversity and Ecosystem Dynamics, University of Amsterdam, Amsterdam, Netherlands

**Keywords:** *C. parapsilosis* species complex, Iran, AFLP genotyping, AFST, *C. orthopsilosis*, *C. metapsilosis*, clonality

## Abstract

Despite the increasing occurrence of *Candida orthopsilosis* and *Candida metapsilosis* in clinical settings, little is known about their microbiological and clinical properties. Herein, we conducted a national retrospective study (2014–2019) from multiple centers in Iran. Among the 1,770 *Candida* isolates collected, we identified 600 *Candida parapsilosis* species complex isolates. Isolate identification was performed by 9-plex PCR, matrix-assisted laser desorption-time of flight mass spectrometry (MALDI-TOF MS), and rDNA sequencing, and antifungal susceptibility testing (AFST) followed CLSI M27-A3/S4; genotyping was performed by amplified fragment length polymorphism (AFLP) analysis; and clinical information was mined. Thirty-one isolates of *C. orthopsilosis* from various clinical sources, one mixed sample (blood) concurrently containing *C. orthopsilosis* and *C. parapsilosis* and one isolate of *C. metapsilosis* from a nail sample were identified. Although both 9-plex PCR and MALDI-TOF successfully identified all isolates, only 9-plex PCR could identify the agents in a mixed sample. For the *C. orthopsilosis* isolates, resistance (non-wild type) was noted only for itraconazole (*n* = 4; 12.5%). Anidulafungin and fluconazole showed the highest and voriconazole had the lowest geometric mean values. AFLP analysis showed three main and four minor genotypes. Interestingly, 90% of nail isolates clustered with 80% of the blood isolates within two clusters, and four blood isolates recovered from four patients admitted to a hospital clustered into two genotypes and showed a high degree of similarity (>99.2%), which suggests that *C. orthopsilosis* disseminates horizontally. Supported by our data and published case studies, *C. orthopsilosis* and *C. metapsilosis* can be linked to challenging clinical failures, and successful outcomes are not always mirrored by *in vitro* susceptibility. Accordingly, conducting nationwide studies may provide more comprehensive data, which is required for a better prognosis and clinical management of patients.

## Introduction

With advancements in identification tools and changes in clinical practices, a distinct trend of an increasing prevalence of non-*Candida albicans Candida* (NCAC) species in clinical settings has been revealed (Lamoth et al., 2018). The recent arrival and increase in the amount of azole-resistant *Candida parapsilosis* isolates (Grossman et al., 2015; Govender et al., 2016; Asadzadeh et al., 2017a; Choi et al., 2018; Thomaz et al., 2018; Singh et al., 2019), and the ability of this species to be horizontally transmitted from the hands of healthcare workers (HCWs) (Thomaz et al., 2018) emphasize the importance of surveillance studies to limit its spread in healthcare settings. Additionally, if left undetected, this yeast can be the source of fatal candidemia outbreaks, and it can persist in the hospital environment for a long period of time (Wang et al., 2016). Phylogenetic analysis performed by Tavanti et al. (2005) showed that *C. parapsilosis* is a species complex comprising *C. parapsilosis* sensu stricto, *C. orthopsilosis*, and *C. metapsilosis* (Tavanti et al., 2005). Although *C. orthopsilosis* and *C. metapsilosis* are less virulent than *C. parapsilosis*, they have the ability to cause a wide range of clinical manifestations ranging from superficial (Feng et al., 2012) to fatal invasive bloodstream infections (Barbedo et al., 2015). Besides, clinical failure for infections caused by *C. orthopsilosis* and *C. metapsilosis* have been reported in some studies, while infected patients underwent prolonged administration of antifungals (Choi et al., 2010; Wessel et al., 2013; Oliveira et al., 2014; Heslop et al., 2015; Charsizadeh et al., 2018). On the other hand, a survey conducted in Italy (Pisa and Rome) showed that 40% of *C. orthopsilosis* isolates were resistant to fluconazole (FLZ), and among them, 100 and 68.7% of FLZ-resistant *C. orthopsilosis* isolates were cross-resistant to two and three most commonly used azoles (Rizzato et al., 2018). Lines of evidence show that azole resistance in the *C. parapsilosis* complex species is mainly mediated by a specific mutation in *ERG11* (A395T) (Choi et al., 2018; Rizzato et al., 2018), and unlike other *Candida* species, efflux pumps might not play a main role in azole resistance (Mello et al., 2017). Moreover, it has been shown that mutations in hotspot 1 (HS1) and HS2 of *FKS1* are linked to echinocandin resistance in the *C. parapsilosis* species complex (Garcia-Effron et al., 2008).

Variability in virulence factors and antifungal susceptibility patterns among members of the *C. parapsilosis* species complex points to the importance of correct species-level identification (Neji et al., 2017a). Phenotypic assays, such as biochemical assays, are unable to differentiate species within the *C. parapsilosis* species complex (Neji et al., 2017b), while PCR-based molecular assays (Tavanti et al., 2007; Mirhendi et al., 2010; Arastehfar et al., 2018), matrix-assisted laser desorption-time of flight mass spectrometry (MALDI-TOF MS) (De Carolis et al., 2014), and sequencing of so-called barcoding genes (Tavanti et al., 2005) allow correct species-level identification.

Genomic studies have led to the discovery that *C. orthopsilosis* and *C. metapsilosis* were derived from the hybridization of species with non-pathogenic lineages (Pryszcz et al., 2014, 2016). As a result, genotyping techniques may provide a better understanding of the evolution of the mechanism of pathogenicity in this complex. Moreover, the application of typing techniques may not only aid in detecting the source of infection but may also broaden our knowledge of the biological niches of a species of interest. Amplified fragment length polymorphism (AFLP) analysis is regarded as the preferred typing choice for members of the *C. parapsilosis* species complex (Tavanti et al., 2007), *Candida albicans* (Asadzadeh et al., 2017b)*, Candida auris* (Schelenz et al., 2016), and *Aspergillus terreus* (Kathuria et al., 2015).

Herein, we conducted a multicenter study and collected all presumptively identified isolates of *C. parapsilosis* from three main metropolitan cities of Iran (Tehran, Shiraz, and Mashhad) from 2014 to 2019. Isolates were identified by MALDI-TOF MS, a previously described 9-plex PCR (Arastehfar et al., 2018) and sequencing of rDNA. Moreover, isolates were genotyped by AFLP, their antifungal susceptibility pattern was determined, and genes conferring resistance to FLZ (*ERG11*) and echinocandins (HS1 and HS2 of *FKS1*) were sequenced.

## Materials and Methods

### Study Design, Ethical Approval, and Growth Conditions

In total, we collected 600 presumptively identified *C. parapsilosis* species complex isolates among 1,770 isolates of *Candida* species isolates recovered (2014–2019) from three major clinical centers in Iran, namely, Tehran, Shiraz, and Mashhad (Table 1). *C. parapsilosis* species complex isolates constituted 33.8% of all *Candida* species isolates recovered from the aforementioned centers. *C. parapsilosis* species complex strains were mainly isolated from blood (*n* = 167) and other non-sterile sites (*n* = 433; Table 1). Studies undertaken by the centers included in this study were individually reviewed and approved by ethical committee members in each center (IR.SUMS.REC.1397.365, IR MUMS fm REC.1397.268, IR. TUMS.SPH.REC.1396.4195). To ensure anonymity, patients were assigned numerical codes. All patients gave written informed consent in accordance with the ethical permit of the centers involved in this study. Strains were grown on Sabouraud dextrose agar (SDA) and incubated at 37°C for 24–48 h. To ensure that samples with mixed species were identified, all clinical samples were struck on CHROMagar (Candiselect, Bio-Rad, USA), and incubated at 37°C for 48 h.

**Table 1:** Presumptively identified *C. parapsilosis* species complex isolates collected from clinical centers.

**Source**	**City**
Blood (*n* = 167)	Tehran, Shiraz, and Mashhad
Vagina (*n* = 100)	Tehran, Shiraz, and Mashhad
Urine (*n* = 80)	Tehran, Shiraz, and Mashhad
Nail (*n* = 80)	Tehran, Shiraz, and Mashhad
Stool (*n* = 40)	Shiraz and Mashhad
Trachea (*n* = 30)	Tehran, Shiraz, and Mashhad
CVC (*n* = 26)	Tehran, Isfahan, and Shiraz
Sputum (*n* = 20)	Tehran, Shiraz, and Mashhad
Throat (*n* = 20)	Tehran, Shiraz, and Mashhad
Skin (*n* = 20)	Tehran, Shiraz, and Mashhad
Ear (*n* = 10)	Tehran, Isfahan, and Mashhad
BALF (*n* = 5)	Isfahan, Shiraz, and Mashhad
Interdigital (*n* = 1)	Tehran
Groin (*n* = 1)	Tehran

*CVC, Central venous catheter; BALF, Bronchoalveolar lavage fluid*.

### DNA Extraction and Identification Strategy

A previously CTAB-based DNA extraction protocol was used to extract DNA samples (Theelen et al., 2001). Primarily, isolated strains were identified by MALDI-TOF MS (MicroFlex LTD, Bruker, Bremen, Germany) using a full-extraction method (Cendejas-Bueno et al., 2012) and a 9-plex PCR differentiating nine species within the *C. albicans, Candida glabrata*, and *C. parapsilosis* species complexes (Arastehfar et al., 2018). Strains identified as *C. orthopsilosis* and *C. metapsilosis* were further identified by sequencing of the large subunit (LSU) and internal transcribed spacer sequences (ITS) of the rDNA domain using LR5 and ITS5 primers (Stielow et al., 2015).

### Genotypic Diversity Using AFLP

To assess the genotypic diversity of *C. orthopsilosis* and *C. metapsilosis*, a previously defined AFLP protocol was used (Marchetta et al., 2018). In brief, 5 μl of a DNA sample was mixed with restriction-ligation reactions containing HpyCH4 IV and *MseI* adapters and restriction enzymes and T4 ligase and incubated at room temperature for 90 min. Subsequently, the ligation-restriction reactions were stopped by the addition of 80 μl of 10 mM Tris-HCl (pH 8.3), and diluted products were added to PCRs containing HpyCH4 IV and *MseI* primers. In the next stage, PCR products were purified using Sephadex (Sigma Aldrich, St. Louis, Missouri, USA) and diluted 50 times with Milli-Q water; 1 μl of PCR product was mixed with master mixes containing standard ladder size, incubated for 1 min at 100°C, and finally subjected to an ABI 3730XL DNA analyzer (Thermo Fisher Scientific, Waltham, Massachusetts, USA). BioNumerics software V7.6 (Applied Math Inc., Austin, Texas, USA) was used to analyze the AFLP data. Reference and type strains of *C. metapsilosis* (CBS 2315, CBS 2916, and CBS 10907) and *C. orthopsilosis* (CBS 10906) were used for comparative purposes.

### Antifungal Susceptibility Testing (AFST)

CLSI M27-A3/S4 broth micro dilution (BMD) was used for the AFST of the *C. orthopsilosis* and *C. metapsilosis* isolates (Clinical Laboratory Standards Institute, 2008, 2012). AFST included the following drugs: fluconazole (FLZ) (Pfizer, New York, USA), voriconazole (VRZ) (Pfizer, New York, USA), itraconazole (ITZ) (Santa Cruz Biotech, Dallas, USA), amphotericin B (AMB) (Sigma Chemical Corporation, St. Louis, MO), micafungin (MFG) (Astellas Pharma Inc., Japan), and anidulafungin (AFG) (Pfizer A/S, Ballerup, Denmark). Reference strains of *C. parapsilosis* (CBS 604) and *Candida krusei* (CBS 5147) were used for quality control purposes. The MIC values were visually determined after incubating the plates for 24 h at 37°C. Due to the lack of a species-specific clinical breakpoint and epidemiological cut-off values for *C. orthopsilosis* and *C. metapsilosis*, the obtained MIC values were compared with those of *C. parapsilosis*. Moreover, due to the inter laboratory variation and unreliability of caspofungin (Espinel-Ingroff et al., 2013), this drug was not investigated in the current study. Isolates showing MIC values ≥8 μg/ml for FLZ, MFG, and ANF and those showing MIC values ≥1 for VRZ were regarded as resistant (Pfaller and Diekema, 2012). Due to the lack of clinical breakpoints for AMB and ITZ, their corresponding MIC values were interpreted based on epidemiological cut-off values (ECV) and non-wild type (NWT) values when the MIC values were >2 and >0.5 μg/ml, respectively (Pfaller and Diekema, 2012).

### PCR and Sequencing of *ERG11* and HS1 and HS2 of *FKS1*

As resistance to azoles in *C. orthopsilosis* is mainly mediated by a specific point mutation (A395T) that resulted in a missense mutation of Y132F (Mello et al., 2017; Rizzato et al., 2018), primers targeting this region were used (Table 2). Moreover, *C. parapsilosis* species complex universal primers (from unpublished data) targeting HS1 and HS2 of *FKS1* (Table 2) were included to explore the potential non-synonymous mutations conferring resistance to echinocandins.

**Table 2:** List of primers used for PCR amplification and sequencing of target genes.

**Oligo name**	**Sequence**	**Target gene/purpose**	**PCR product sizes (bp)**	**Reference**
*FKS1*-HS1-F	CATACRTTTACTGCAAACTTTGT	Cp*FKS1*/PCR and sequencing	417	Unpublished data
*FKS1*-HS1-R	GATTTCCATTTCGGTGGT	Cp*FKS1*/PCR and sequencing	417	Unpublished data
*FKS1*-HS2-F	TGCATRTGAACGAAGATATTTA	Cp*FKS1*/PCR and sequencing	568	Unpublished data
*FKS1*-HS2-R	GCAACAAARACTTCAAACAT	Cp*FKS1*/PCR and sequencing	568	Unpublished data
*ERG11*-F	ATGGCATTAGTTGACTTA	Cp*ERG11*/PCR and sequencing	495	This study
*ERG11*-R	TCTCCTCTAATCAACGGA	Cp*ERG11*/PCR and sequencing	495	This study

PCRs contained the following ingredients: 5 μl of PCR buffer (10X NH_4_ without MgCl2), 2 mM MgCl2, 10 picomole target primers (*ERG11*F/R and HS1F/R and HS2F/R), 0.2 mM mixed dNTPs (dNTP mix, 100 mM, Bioline), and 1.25 units of *Taq* polymerase (BioTaq DNA Polymerase, Bioline). Milli-Q water was used to adjust the volume to 50 μl. PCR reactions were subjected to Applied Biosystem 2720 Thermal Cycler (Thermo Fisher Scientific, Waltham, Massachusetts, USA) with the following program: one cycle of 95°C for 5 min; followed by 35 cycles of 95°C for 30 s, 52°C for 30 s, and 72°C for 30 s; and finally, one cycle of 72°C for 8 min.

The dideoxy-chain termination sequencing protocol was used for sequencing of target genes, and the generated contigs were curated, assembled and edited by SeqMan Pro (DNASTAR, Madison, USA). Curated sequences were aligned using MEGA v7.0 (Temple University, Philadelphia, USA). The obtained sequences of *ERG11* were compared with the corresponding reference sequences of XM_003870254.1 (Riccombeni et al., 2012; Rizzato et al., 2018), and the sequences of HS1 and HS2 were compared with those presented by Garcia-Effron et al. (2008).

### Deposition of *C. orthopsilosis* and *C. metapsilosis* Strains and Corresponding Accession Numbers

The *C. orthopsilosis* and *C. metapsilosis* strains obtained from this study were deposited in the culture collection of Westerdijk Fungal Biodiversity Institute, and their corresponding sequences of ITS and LSU rDNA, HS1 and HS2 of *FKS1*, and *ERG11* were deposited in GenBank (https://www.ncbi.nlm.nih.gov/genbank/) (Supplementary Table 1).

## Results

### Clinical Profiles

In total, 32 *C. orthopsilosis* isolates were recovered from 31 patients, with a median age of 39 years old (1 month-90 years) and one *C. metapsilosis* isolate from a 30-year-old man (Table 3). Women constituted the vast majority of patients (*n* = 22; 68.7%). Tehran had the highest number of *C. orthopsilosis* isolates (*n* = 19; 57.6%), followed by Mashhad (*n* = 13; 39.4%), and Shiraz (*n* = 1; 3%). *C. orthopsilosis* isolates were mainly from blood (*n* = 10; 31.2%) and nail (*n* = 10; 31.2%) samples, followed by urine (*n* = 5; 15.6%), vaginal (*n* = 3; 9.3%), tracheal (*n* = 2; 6.2%), and groin and interdigital (each *n* = 1; 3.2%) samples (Table 3). The only isolate of *C. metapsilosis* was recovered from a nail sample. Diabetes (*n* = 5), hematological malignancies (*n* = 4), and pneumonia (*n* = 2) were the most encountered underlying conditions (considering that the majority of samples were obtained from outpatients and the underlying conditions were not available for some patients). All patients with invasive candidiasis due to *C. orthopsilosis* were treated with broad-spectrum antibiotics. In total, 10 patients were treated with antifungals and AMB was the most widely used antifungal (*n* = 7; 70%), followed by the combination of CSP, FLZ, and AMB (each *n* = 3; 30%; Table 3). Four patients infected with *C. orthopsilosis* died and the corresponding isolates were recovered from blood (*n* = 2), vagina (*n* = 1), and trachea (*n* = 1). For comparison purposes, case report studies describing microbiological and clinical outcomes are presented in Supplementary Table 2.

**Table 3:** Clinical data obtained from patients positive for *C. orthopsilosis* or *C. metapsilosis*.

**Isolate #**	**Species**	**Age/sex**	**City/hospital/unit**	**Underlying conditions**	**Isolation date**	**Source**	**Antibiotic used**	**Antifungal used**	**Outcome**
TMML385	*C. orthopsilosis*	24/F	Tehran/outpatient	Healthy	2015/04/03	Nail	ND	ND	Survived
TMML397	*C. orthopsilosis*	52/F	Tehran/outpatient	Healthy	2014/02/22	Nail	ND	ND	Survived
TMML399	*C. orthopsilosis*	49/M	Tehran/outpatient	Healthy	2016/12/17	Nail	ND	ND	Survived
TMML406	*C. orthopsilosis*	58/F	Tehran/outpatient	Healthy	2013/09/15	Nail	ND	ND	Survived
TMML407	*C. orthopsilosis*	24/F	Tehran/outpatient	Healthy	2015/02/05	Nail	ND	ND	Survived
TMML414	*C. orthopsilosis*	16/F	Tehran/outpatient	Healthy	2015/11/06	Nail	ND	ND	Survived
TMML415	*C. orthopsilosis*	54/M	Tehran/outpatient	ND	2015/02/03	Interdigital	ND	ND	Survived
TMML430	*C. orthopsilosis*	39/F	Tehran/outpatient	Healthy	2016/10/23	Nail	ND	ND	Survived
TMML443	*C. orthopsilosis*	51/F	Tehran/outpatient	Healthy	2014/10/20	Nail	ND	ND	Survived
TMML454	*C. orthopsilosis*	74/F	Tehran/outpatient	Healthy	2015/09/06	Nail	ND	ND	Survived
TMML456	*C. orthopsilosis*	33/M	Tehran/outpatient	Healthy	2015/02/19	Nail	ND	ND	Survived
TMML464	*C. orthopsilosis*	50/F	Tehran/outpatient	Healthy	2016/12/01	Nail	ND	ND	Survived
N2	*C. orthopsilosis*	35/F	Mashhad/22 Bahman/outpatient	Pregnant/UTI	2018/02/23	Urine	ND	ND	Survived
N5	*C. orthopsilosis*	60/F	Mashhad/Jihad/ND	Diabetes/UTI	2018/01/26	Urine	ND	ND	Survived
N9	*C. orthopsilosis*	34/F	Mashhad/Rajaee/ND	Vaginitis	2018/03/03	Trachea	ND	ND	Survived
N13	*C. orthopsilosis*	70/F	Mashhad/22Bahman/ICU	Diabetes/Pneumonia	2017/11/01	Trachea	Yes	AMB	Died
N14	*C. orthopsilosis*	90/M	Mashhad/22 Bahman/ICU	Diabetes/Pneumonia	2018/12/22	Vagina	Yes	AMB	Died
N19	*C. orthopsilosis*	40/F	Mashhad/Jihad 2/outpatient	Vaginitis	2018/04/22	Urine	ND	ND	Survived
N20	*C. orthopsilosis*	45/F	Mashhad/Fajr/outpatient	Diabetes/UTI	2018/05/05	Urine	ND	ND	Survived
N27	*C. orthopsilosis*	33/F	Mashhad/Rajaee/outpatient	UTI	2018/02/23	Urine	ND	ND	Survived
N30	*C. orthopsilosis*	39/F	Mashhad/Jihad/outpatient	UTI	2017/12/01	Vagina	ND	ND	Survived
N31	*C. orthopsilosis*	40/F	Mashhad/Arya/outpatient	Vaginitis	2018/01/01	Nail	ND	ND	Survived
N232	*C. metapsilosis*	30/M	Mashhad/Imam Reza/outpatient	Healthy	2017/01/05	Nail	ND	ND	Survived
Mir 147	*C. orthopsilosis*	3/F	Tehran/Children's Medical Center/NICU	ALL	2015/01/27	Blood	Yes	AMB	Survived
Mir 187	*C. orthopsilosis*	3/F	Tehran/Children's Medical Center/PICU	ALL	2014/12/24	Blood	Yes	AMB+CAS	Survived
Mir 496	*C. orthopsilosis*	8/M	Tehran/Children's Medical Center/PICU	Hyper-IgM syndrome	2015/11/21	Blood	Yes	AMB+CAS	Survived
Mir 606	*C. orthopsilosis*	1M^**A**^/M	Tehran/Children's Medical Center/NICU	Prematurity	2016/06/01	Blood	Yes	AMB+FLZ	Survived
Mir 617	*C. orthopsilosis*	1/F	Tehran/Children's Medical Center/ Immunology NICU	Immunodeficiency	2016/06/15	Blood	Yes	AMB	Survived
Mir 618	*C. orthopsilosis*	7/M	Tehran/Children's Medical Center/PICU	Lymphoma	2016/06/20	Blood	Yes	AMB+FLZ	Survived
48BC	*C. orthopsilosis*	16/M	Tehran/Imam Khomeini/Endocrinology	T Cell ALL, AML	2018/05/13	Blood	Yes	FLZ+AMB+CAS	Survived
N1R	*C. orthopsilosis*	40/M	Mashhad/22 Bahman/ICU	Diabetes	2017/12/16	Blood	Yes	FLZ	Died
N114	*C. orthopsilosis*	48/M	Mashhad/Imam Reza/ICU	PTE	2017/02/08	Blood	Yes	None	Died
SU-236	*C. orthopsilosis*	1/F	Shiraz/Namazi/ICU	Bowel obstruction	2017/08/06	Blood	Yes	None	Survived

*ND, No data; ALL, Acute lymphocytic leukemia; AML, Acute myeloid leukemia; PTE, Pulmonary thromboembolism; F, Female; M, Male; IgM, Immunoglobulin M; AMB, Amphotericin B; FLZ, Fluconazole; CAS, Caspofungin; UTI, Urinary tract infection. A, M, Month*.

### Identification

**Figure 1:**
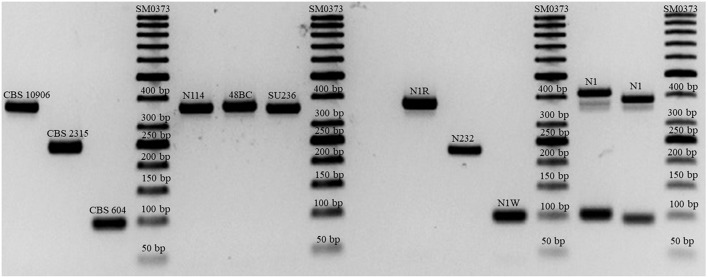
Successful differentiation of the *C. parapsilosis* species complex and mixed isolates of *C. parapsilosis* and *C. orthopsilosis* (N1 with double bands representing both species).

*C. orthopsilosis* and *C. metapsilosis* comprised 1.8 and 0.05% of all *Candida* species isolates and 5.3 and 0.17% of all *C. parapsilosis* species complex isolates, respectively. Both 9-plex PCR and MALDI-TOF MS consistent with ITS and LSU rDNA sequencing successfully identified all *C. orthopsilosis, C. metapsilosis*, and *C. parapsilosis* isolates. One of the blood samples concurrently harbored both *C. orthopsilosis* and *C. parapsilosis*, which were identified based on colony morphology (wrinkled colonies for *C. parapsilosis* and round colonies for *C. orthopsilosis*). Sequencing and MALDI-TOF MS identified this mixed sample as *C. parapsilosis*, while the 9-plex PCR successfully identified both *C. parapsilosis* and *C. orthopsilosis* (Figure 1).

### Genotypic Diversity Using AFLP

**Figure 2:**
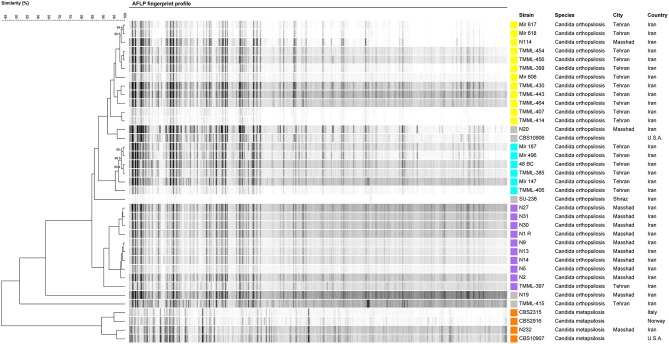
AFLP fingerprint profile of *C. orthopsilosis* and *C. metapsilosis* isolates included in this study. Each genotype is assigned a distinct color.

AFLP was employed to explore the genotypic diversity of *C. orthopsilosis* and *C. metapsilosis* isolates included in this study (Figure 2). In total, three major genotypes, namely, G1 (*n* = 12), G2 (*n* = 6), and G3 (*n* = 10), along with four minor genotypes, each containing one strain, were detected. Isolates of G1 were mainly obtained from nail samples (66.6%), and 80% of blood isolates (*n* = 8) belonged to G1 and G2 clustered with isolates recovered from nail samples (*n* = 10; 90%; Figure 2). Isolates grouped in G3 were from a diverse range of clinical sources, including nails, blood, urine, vagina, trachea, and interdigital. A geographical trend was observed for the clustering of some genotypes, where G1 and G3 isolates came mainly from Tehran and Mashhad, respectively. Moreover, four blood isolates distributed in G1 and G2 (each containing two isolates) recovered from a neonatal ICU ward in Tehran (Children's Medical Center) showed a clonal pattern with a similarity of >99.2% (Figure 2).

### Antifungal Susceptibility Pattern

Antifungal susceptibility data for all isolates of *C. orthopsilosis* and *C. metapsilosis* are presented in Tables 4, 5. All isolates were susceptible to ANF (≤8 μg/ml) and MFG (≤8 μg/ml) and had a wild-type (WT) phenotype in the presence of AMB (<2 μg/ml). FLZ-susceptible dose-dependent (SDD) (= 4 μg/ml) and VRZ-intermediate (I) (0.25–0.5 μg/ml) were noted in 3.12 and 6.25% of isolates, respectively. For ITZ, 12.5% of isolates showed a NWT phenotype against this drug (>0.5 μg/ml). ANF and FLZ showed the highest geometric mean values (~1.0), followed by MFG (0.68), ITZ and AMB (0.31), and VRZ (0.02) (Table 4).

**Table 4:** Antifungal susceptibility data derived from *C. orthopsilosis* isolates in this study.

**Antifungal drugs**	**MIC Values**													**Range**	**GM**	**MIC 50**	**MIC 90**
	**≤0.015**	**0.03**	**0.06**	**0.125**	**0.25**	**0.5**	**1**	**2**	**4**	**8**	**16**	**32**	**≥64**				
FLZ				1	10	8	5	8	1					0.125–4	1.03	0.5	2
VRZ	15	10	6		1	1								≤0.0.15–0.5	0.02	0.03	0.06
ITZ		4	5	3	9	8	2	2						0.03–2	0.31	0.25	1
MFG		6	4	1	6	11	5							0.03–1	0.68	0.5	1
ANF			2	3	7	9	11	1						0.06–2	1.05	0.5	1
AMB	5	2	8	2	8	6	2							≤0.0.15–1	0.312	0.125	0.5

*GM, Geometric mean value; FLZ, Fluconazole; VRZ, Voriconazole; ITZ, Itraconazole; MFG, Micafungin; ANF, Anidulafungin; AMB, Amphotericin B; and MIC, Minimum inhibitory concentration*.

**Table 5:** Antifungal susceptibility testing data and sequencing of genes conferring resistance to echinocandins (HS1 and HS2 of *FKS1*) and azoles (*ERG11*).

**Patient #**	**Species**	**Genotype**	**MIC values (μg/ml)**					
			**FLZ**	**VRZ**	**ITZ**	**MCF**	**ANF**	**AMB**
TMML385	*C. orthopsilosis*	G2	1	0.015	0.125	0.03	0.06	0.015
TMML397	*C. orthopsilosis*	G3	2	0.03	0.5	0.25	0.25	0.015
TMML399	*C. orthopsilosis*	G1	2	0.03	0.25	0.03	0.25	0.015
TMML406	*C. orthopsilosis*	G2	2	0.03	0.5	0.06	0.125	0.06
TMML407	*C. orthopsilosis*	G1	2	0.03	0.25	0.03	0.25	0.06
TMML414	*C. orthopsilosis*	G1	2	0.03	0.5	0.03	0.25	0.25
TMML415	*C. orthopsilosis*	SG	1	0.015	0.25	0.06	0.5	0.06
TMML430	*C. orthopsilosis*	G1	2	0.015	0.5	0.06	0.25	0.015
TMML443	*C. orthopsilosis*	G1	4	0.015	2	0.03	0.06	0.03
TMML454	*C. orthopsilosis*	G1	2	0.015	1	0.125	0.125	0.03
TMML456	*C. orthopsilosis*	G1	1	0.015	1	0.03	0.125	0.06
TMML464	*C. orthopsilosis*	G1	2	0.015	2	0.06	0.25	0.015
N1R	*C. orthopsilosis*	G3	0.25	0.015	0.5	1	1	0.5
N2	*C. orthopsilosis*	G3	1	0.5	0.5	1	1	1
N5	*C. orthopsilosis*	G3	0.25	0.015	0.06	0.5	0.5	0.06
N9	*C. orthopsilosis*	G3	0.5	<0.015	0.03	0.5	0.5	0.25
N13	*C. orthopsilosis*	G3	0.25	<0.015	0.03	0.5	1	0.125
N14	*C. orthopsilosis*	G3	0.25	0.015	0.25	1	1	0.125
N19	*C. orthopsilosis*	SG	0.25	0.03	0.125	1	0.5	0.5
N20	*C. orthopsilosis*	SG	0.5	0.06	0.5	0.5	0.25	0.25
N27	*C. orthopsilosis*	G3	0.25	<0.015	0.03	0.5	1	1
N30	*C. orthopsilosis*	G3	0.25	0.015	0.125	0.5	1	0.06
N31	*C. orthopsilosis*	G3	0.5	0.06	0.06	0.5	0.5	0.25
N114	*C. orthopsilosis*	G1	0.25	0.06	0.03	0.5	2	0.06
N232	*C. metapsilosis*	SG	1	<0.015	0.06	1	1	0.5
Mir147	*C. orthopsilosis*	G2	0.25	0.06	0.25	0.25	0.5	0.5
Mir187	*C. orthopsilosis*	G2	0.5	0.03	0.25	0.25	0.5	0.5
Mir496	*C. orthopsilosis*	G2	0.5	0.06	0.25	0.25	0.5	0.5
Mir606	*C. orthopsilosis*	G1	0.5	0.06	0.5	0.5	1	0.5
Mir617	*C. orthopsilosis*	G1	0.5	0.03	0.5	0.25	0.5	0.25
Mir618	*C. orthopsilosis*	G1	0.5	0.25	0.25	1	1	0.06
48BC	*C. orthopsilosis*	G2	0.25	0.03	0.06	0.5	1	0.25
SU-236	*C. orthopsilosis*	SG	0.125	<0.015	0.25	0.5	1	0.25

*G, Genotype; SG, Single genotype; NSD, No sequence data*.

### PCR and Sequencing of *ERG11* and HS1 and HS2 of *FKS1*

Although successful PCR amplification and sequencing results were obtained for all target genes of *C. orthopsilosis*, sequences of acceptable quality were not obtained for *ERG11* of *C. metapsilosis*. All isolates harbored WT *ERG11* and HS1 and HS2 of *FKS1* (Table 5).

## Discussion

In this study, we present the largest collection of *C. orthopsilosis* (*n* = 32) and the first case of *C. metapsilosis* recovered from Iranian patients. In a previous study, Mohammadi et al. (2017) explored the antifungal susceptibility of different and smaller sets of Iranian *C. orthopsilosis* (*n* = 18) isolates, but the association of genotypic diversity and clinical data, mechanism of resistance via sequencing of *ERG11* and HS1 and HS2 of *FKS1*, and comparison of MALDI-TOF and 9-plex PCR in the context of sequencing were not assessed.

### Geographical-Dependent Variation in Prevalence Is Associated With Strain-Dependent Virulence Attributes and Commensal and Environmental Microbiome Communities

In our study, *C. orthopsilosis* and *C. metapsilosis* were responsible for 5.3 and 0.17% of *C. parapsilosis* species complex infections, respectively. The extremely low prevalence of *C. metapsilosis* in this study is similar to observations from other studies conducted in Iran (Mohammadi et al., 2017), Italy (Romeo et al., 2012; Lovero et al., 2016), Venezuela (Moreno et al., 2017), and Kuwait (Asadzadeh et al., 2009) but, contrasts the observations reported for East China (Ge et al., 2012). In contrast, other studies from Africa (Neji et al., 2017b), Latin America (Goncalves et al., 2010), Europe (Gomez-Lopez et al., 2008), and other Asian countries (Tay et al., 2009; Chen et al., 2010) isolated both *C. orthopsilosis* and *C. metapsilosis* from blood samples, although with varying prevalences. The low prevalence of *C. metapsilosis* could be related to the reduced virulence and biofilm-production ability of this emerging pathogen (Gago et al., 2014), but this substantial variability might be indicative of the involvement of other factors, such as variation in the microbiome structure observed in different populations and environments. For instance, in East China, authors noted that *C. metapsilosis* was responsible for 60% of the *C. parapsilosis* species complex infections in one of the centers included in the study, and these isolates were mainly obtained from cutaneous samples of dermatological outpatients (Ge et al., 2012). The authors attributed this unusual *C. metapsilosis* prevalence to a different microbiome population of infected patients who might have shared the same working environment (Ge et al., 2012). This might be a plausible explanation, as *C. metapsilosis* has been found in the commensal (Ghannoum et al., 2010) and environmental (Trofa et al., 2008) microbiomes. Additionally, it has been shown that drinking water (Willis et al., 2018) and specific lifestyle (Valles et al., 2018) might have an impact on the microbiome structure, and this finding may further justify this observed marked difference in the epidemiology of this species complex.

### Probable Clonal Expansion of *C. orthopsilosis* in Healthcare Settings

Although *C. parapsilosis* is one of the most prominent *Candida* species to cause clonal outbreaks (Wang et al., 2016; Singh et al., 2019), this phenomenon has not been observed for *C. orthopsilosis* and *C. metapsilosis*. Interestingly, we noted that four isolates obtained from four patients in a neonatal ICU ward (Tehran) clustered in two genotypes with a high degree of genetic similarity (>99.2%), which is in contrast to the observation that clinical *C. orthopsilosis* isolates showed a high level of genetic diversity (Tavanti et al., 2007). The hybrid nature of *C. orthopsilosis* isolates (Pryszcz et al., 2014) and the fact that those isolates were recovered from various health care settings located in different countries (Tavanti et al., 2007) might explain the high level of genetic diversity observed in that study. On the other hand, we noticed that 80% of *C. orthopsilosis* blood isolates clustered with 90% of *C. orthopsilosis* isolates obtained from nail samples. This finding, along with the possible clonality of *C. orthopsilosis* isolates and the simultaneous isolation of this species from both central venous catheter (CVC) and blood samples reported previously (Barbedo et al., 2015), might imply that *C. orthopsilosis*, similar to *C. parapsilosis*, could be horizontally transferred from the hands of healthcare workers.

### MALDI-TOF MS and Sequencing Failed to Identify Mixed Isolates Containing *C. parapsilosis* and *C. orthopsilosis*

MALDI-TOF MS and Sanger sequencing are the most accurate means of identification in clinical settings. However, in this study, we observed that both MALDI-TOF MS and sequencing of ITS and LSU rDNA failed to identify *C. parapsilosis* and *C. orthopsilosis* from a mixed isolate obtained from blood, while the 9-plex PCR yielded two bands representing both species. A study from Portugal showed that 9.5% of *C. parapsilosis* blood isolates were a mixture of *C. parapsilosis* and *C. orthopsilosis* (Barbedo et al., 2015). Because polyfungal infections are associated with a high rate of mortality (Kim et al., 2013), it seems relevant to utilize sensitive and specific assays to identify the causative agents of mixed samples. Moreover, the application of such techniques can reveal a possible mixed sample to technicians and, as a result, might prevent the underestimation of these emerging yeast species; consequently, this may lead to a better epidemiological, microbiological, and clinical understanding.

### High Rate of ITZ-NWT Phenotype for *C. orthopsilosis* Isolates

Except for 3.12% FLZ-SDD, 6.25% VRZ-I, and 12.5% ITZ-NWT phenotypes, our *C. orthopsilosis* isolates together with a single *C. metapsilosis* isolate were susceptible to all major antifungal drugs tested. The lack of FLZ and echinocandin resistance was further proven by sequencing *ERG11* and HS1 and HS2 of *FKS1*. Although antifungal resistance for *C. orthopsilosis* (Mohammadi et al., 2017; Brilhante et al., 2018) and *C. metapsilosis* (Chen et al., 2010) is considered a rare phenomenon, a study conducted in Italy revealed that almost 40% of *C. orthopsilosis* isolates were resistant to FLZ, and among them, almost 100% of isolates were cross-resistant to at least two azole drugs (Rizzato et al., 2018). Given that some FLZ-R genotypes of *C. parapsilosis* can persist in hospital settings for several years (Choi et al., 2018) in addition to the possible clonality of *C. orthopsilosis* presented in this study, this finding emphasizes the paramount importance of typing studies to limit the spread and to find the source of a given *C. orthopsilosis* FLZ-R genotype.

### High Rate of Clinical Failure and Discrepancy Between *in vitro* Susceptibility Testing and Clinical Outcome

*C. orthopsilosis* followed by *C. metapsilosis* are considered the least virulent and benign species within the *C. parapsilosis* species complex, while studies dealing with clinical cases proved otherwise and showed that these two species can be linked to challenging septic arthritis (Heslop et al., 2015), keratitis (Wessel et al., 2013), and blood-borne infections (Choi et al., 2010; Oliveira et al., 2014; Charsizadeh et al., 2018). In our study, almost 33% of patients admitted to the ICU (*n* = 4) died, despite three of them received AMB or FLZ. Surprisingly, the MIC values of those *C. orthopsilosis* isolates derived from treated patients were susceptible to all antifungals used (except for one ITZ-R isolate). This discrepancy between clinical outcome and *in vitro* AFST has been noted in a keratitis case caused by *C. orthopsilosis* (Wessel et al., 2013). In that study, the recovered *C. orthopsilosis* isolate was susceptible to FLZ, VRZ, and AMB, and despite prolonged treatment with topical or systemic VRZ along with AMB, the patient manifested clinical failure, and surgical intervention finally alleviated the symptoms (Wessel et al., 2013). Surprisingly, apart from one study that showed the efficacy of FLZ (Alencar et al., 2017), the remaining studies unanimously showed the fatality of *C. orthopsilosis* infection (Choi et al., 2010; Oliveira et al., 2014; Charsizadeh et al., 2018) along with the lack of efficacy of FLZ and CSP (Choi et al., 2010), FLZ and AMB (Oliveira et al., 2014), FLZ (Heslop et al., 2015), and AMB (Charsizadeh et al., 2018). This variability in clinical outcome is shown even for the two *C. metapsilosis* fungemia cases, where one study showed successful treatment via only CVC removal without antifungal drug intervention (Asadzadeh et al., 2016), while the other study showed FLZ and AMB treatment failure (Oliveira et al., 2014). In addition to host-related underlying conditions and variability in tissue penetration of antifungal drugs (Zhao et al., 2017), these discrepancies between *in vitro* AFST and clinical outcome and the relatively high rate of clinical failure in case studies could be a strain-dependent phenomenon and may be explained by variation in microbiological factors, such as biofilm formation. Moreover, a recent study disclosed that this discrepancy between clinical outcome and MIC data might be due to the presence of a distinguished category of cells called tolerant cells that typically are miscategorized as susceptible via *in vitro* susceptibility protocols, while these cells can slowly grow in the presence of antifungal protocols (Rosenberg et al., 2018).

## Conclusion

The discrepancy between *in vitro* AFST and the clinical failure of infections caused by both *C. orthopsilosis* and *C. metapsilosis* underscores the importance of the implementation of appropriate identification tools. Although MALDI-TOF MS and Sanger sequencing are the most accurate means of identification currently used in medical mycology, the application of molecular assays for laboratories lacking these tools is recommended to broaden our knowledge about the epidemiology, clinical profile, and microbiological features of these two underrated *Candida* species. However, the application of molecular assays, such as 9-plex PCR, can be a supplementary tool to guide the identification of causative agents of mixed samples that are not identifiable via CHROMagar and even MALDI-TOF MS and rDNA sequencing. Moreover, the possible clonal transmission of *C. orthopsilosis* noted in this study warrants further analysis to reinforce our findings and may reveal that employing resolutive typing techniques may have infection control implications in the case of outbreaks caused by *C. orthopsilosis*. Unfortunately, the lack of isolates derived from environmental samples and hands of healthcare workers from hospitals where *C. orthopsilosis* blood isolates were obtained and the lack of assessment of the biofilm-production ability of *C. orthopsilosis* isolates are the main limitations of this study.

## Data Availability

The raw data supporting the conclusions of this manuscript will be made available by the authors, without undue reservation, to any qualified researcher.

## Ethics Statement

Studies undertaken by involved centers were individually reviewed and approved by ethical committee members in each center (IR.SUMS.REC.1397.365, IR MUMS fm REC.1397.268, IR. TUMS.SPH.REC.1396.4195). In order to ensure the anonymity, patients were assigned with numerical codes. All patients gave written informed consent as in accordance with the ethical committee of centers involved.

## Author Contributions

AA, SK, and FD designed the study, collected the data, drafted the manuscript, and performed part of the experimental studies. M-JN, SM, AC, M-RS, HZ, AR, SD, ZZ, and FH participated in the experimental studies, data collection, and revising of the manuscript. SK, M-JN, AC, and KZ provided the clinical isolates. WP, KZ, and TB supervised the study and revised the manuscript.

### Conflict of Interest Statement

The authors declare that the research was conducted in the absence of any commercial or financial relationships that could be construed as a potential conflict of interest.
