# Supplementary material for: Molecular Identification, Genotypic Diversity, Antifungal Susceptibility, and Clinical Outcomes of Infections Caused by Clinically Underrated Yeasts, Candida orthopsilosis, and Candida metapsilosis: An Iranian Multicenter Study (2014–2019)
Source: Front Cell Infect Microbiol. 2019 Jul 30;9:264. doi: 10.3389/fcimb.2019.00264 (PMC6682699; doi:10.3389/fcimb.2019.00264)
Supplement: Supplementary file 1 [file Table_1.docx]

Supplementary Table 1. CBS number of *C. orthopsilosis* and *C. metapsilosis* strains in this study and their corresponding accession numbers to ITS and LSU rDNA loci, *ERG11* and HS1 and HS2 of *FKS1*

| **Isolate #** | **Species** | **CBS #** | **ITS accession #** | **LSU accession #** | **HS1/*FKS1* accession #** | **HS2/*FKS1* accession #** | ***ERG11* accession #** |
| --- | --- | --- | --- | --- | --- | --- | --- |
| TMML385 | *C. orthopsilosis* | CBS 15880 | MK561065 | MK560801 | MK532238 | MK532242 | MK585276 |
| TMML397 | *C. orthopsilosis* | CBS 15881 | MK561066 | MK560802 | MK532239 | MK532243 | MK585277 |
| TMML399 | *C. orthopsilosis* | CBS 15882 | MK561067 | MK560803 | MK532240 | MK532244 | MK585278 |
| TMML406 | *C. orthopsilosis* | CBS 15883 | MK561061 | MK560797 | MK532241 | MK532245 | MK585279 |
| TMML407 | *C. orthopsilosis* | CBS 15904 | MK561060 | MK560796 | MK532246 | MK541910 | MK585280 |
| TMML414 | *C. orthopsilosis* | CBS 15884 | MK561068 | MK560804 | MK532247 | MK541911 | MK585281 |
| TMML415 | *C. orthopsilosis* | CBS 15885 | MK561069 | MK560805 | MK532248 | MK541912 | MK585282 |
| TMML430 | *C. orthopsilosis* | CBS 15886 | MK561070 | MK560806 | MK532249 | MK541913 | MK585283 |
| TMML443 | *C. orthopsilosis* | CBS 15887 | MK561062 | MK560798 | MK532250 | MK541914 | MK585284 |
| TMML454 | *C. orthopsilosis* | CBS 15888 | MK561063 | MK560799 | MK532251 | MK541915 | MK585285 |
| TMML456 | *C. orthopsilosis* | CBS 15889 | MK561071 | MK560807 | MK532252 | MK541916 | MK585286 |
| TMML464 | *C. orthopsilosis* | CBS 15890 | MK561064 | MK560800 | MK532253 | MK541917 | MK585287 |
| N2 | *C. orthopsilosis* | CBS 15845 | MK561043 | MK560779 | MK576034 | MK576035 | MK585288 |
| N5 | *C. orthopsilosis* | CBS 15846 | MK561044 | MK560780 | MK585310 | MK585326 | MK585289 |
| N9 | *C. orthopsilosis* | CBS 15847 | MK561045 | MK560781 | MK585311 | MK585325 | MK585290 |
| N13 | *C. orthopsilosis* | CBS 15848 | MK561046 | MK560782 | MK585312 | MK585333 | MK585291 |
| N14 | *C. orthopsilosis* | CBS 15849 | MK561047 | MK560783 | MK585313 | MK585332 | MK585292 |
| N19 | *C. orthopsilosis* | CBS 15850 | MK561048 | MK560784 | MK585314 | MK585331 | MK585293 |
| N20 | *C. orthopsilosis* | CBS 15851 | MK561049 | MK560785 | MK585315 | MK585330 | MK585294 |
| N27 | *C. orthopsilosis* | CBS 15852 | MK561050 | MK560786 | MK585316 | MK585329 | MK585295 |
| N30 | *C. orthopsilosis* | CBS 15853 | MK561051 | MK560787 | MK585317 | MK585328 | MK585296 |
| N31 | *C. orthopsilosis* | CBS 15854 | MK561052 | MK560788 | MK585318 | MK585327 | MK585297 |
| N232 | *C. metapsilosis* | CBS 15855 | MK561001 | MK561031 | MK585308 | MK585309 | NSD |
| Mir 147 | *C. orthopsilosis* | CBS 15856 | MK561054 | MK560790 | MK585319 | MK585339 | MK585298 |
| Mir 187 | *C. orthopsilosis* | CBS 15857 | MK561055 | MK560791 | MK585320 | MK585334 | MK585299 |
| Mir 496 | *C. orthopsilosis* | CBS 15858 | MK561056 | MK560792 | MK585321 | MK585336 | MK585300 |
| Mir 606 | *C. orthopsilosis* | CBS 15859 | MK561057 | MK560793 | MK585322 | MK585335 | MK585301 |
| Mir 617 | *C. orthopsilosis* | CBS 15860 | MK561058 | MK560794 | MK585323 | MK585337 | MK585302 |
| Mir 618 | *C. orthopsilosis* | CBS 15861 | MK561059 | MK560795 | MK585324 | MK585338 | MK585303 |
| 48BC | *C. orthopsilosis* | CBS 15892 | MK561072 | MK560809 | MK532239 | MK532243 | MK585303 |
| N1R | *C. orthopsilosis* | CBS 15878 | MK561042 | MK560778 | MK532238 | MK532242 | MK585305 |
| N114 | *C. orthopsilosis* | CBS 15879 | MK561053 | MK560789 | MK532241 | MK532245 | MK585306 |
| SU-236 | *C. orthopsilosis* | CBS 15862 | MK561073 | MK560808 | MK532240 | MK532244 | MK585307 |

ND; No data, ALL; Acute lymphocytic leukemia, AML; Acute myeloid leukemia, PTE; Pulmonary thromboembolism
