# Supplementary material for: Molecular Identification, Genotypic Diversity, Antifungal Susceptibility, and Clinical Outcomes of Infections Caused by Clinically Underrated Yeasts, Candida orthopsilosis, and Candida metapsilosis: An Iranian Multicenter Study (2014–2019)
Source: Front Cell Infect Microbiol. 2019 Jul 30;9:264. doi: 10.3389/fcimb.2019.00264 (PMC6682699; doi:10.3389/fcimb.2019.00264)
Supplement: Supplementary file 2 [file Table_2.docx]

Supplementary Table 2. Clinical and microbiological data obtained from published case reports

| **Reference** | **Outcome** | **T. D** | **Treatment** | **Risk factors** | **Symptoms** | **Sample type** | **Age/sex** | **Resistance** | **Susceptible** | **Species** | **Country**  **)Year)** |
| --- | --- | --- | --- | --- | --- | --- | --- | --- | --- | --- | --- |
| (Choi et al, 2012) | Died | 50 D | FLZ, CAS | Panperitonitis, gastrectomy | - | Blood | 75 Y/M | - | - | *C. orthopsilosis* | Korea 2010 |
| (Wessel et al, 2013) | Treated using heavily administration of antifungals | 2 Mo | AMB+VRZ penetrating keratoplasty | DALK | Fungal keratitis |  | 39 Y/M | AMB, VCZ |  | *C. orthopsilosis* | Germany 2012 |
| (Oliveira et al, 2014) | Died | 5 D | AMB, FLZ | Low birth weight | High fever chills, rapid breathing, rapid heartbeat | Blood | 16 D/- | - | AMB, FLZ | *C. orthopsilosis* | Brazil  2013 |
| (Oliveira et al, 2014) | Died | 5 D | AMB, FLZ | Respiratory problem | High fever chills, rapid breathing, rapid heartbeat | Blood | 10 Mo/- | FLZ-SDD | AMB | *C. orthopsilosis* | Brazil  2013 |
| (Oliveira et al, 2014) | Died | 5 D | AMB, FLZ | Respiratory problem | High fever chills, rapid breathing, rapid heartbeat | Blood | 4 Y/- | FLZ-SDD | AMB | *C. metapsilosis* | Brazil  2013 |
| (Heslop et al, 2015) | Not treated | 12 Mo | FLZ | Systemic lupus erythematosus, corticosteroid therapy, antibiotic therapy | Painful swelling of the left knee | Tissue and joint fluids of the left knee | 28 Y/M | - | AMB, FLZ, ITZ, PSZ, VCZ, KTZ, FLC | *C. orthopsilosis* | Jamaica 2015 |
| (Asadzadeh et al, 2016) | Died of other complications | Clearance by CVC removal | No treatment CVC removal | Neurodegenerative disorder, CVC, mechanical ventilation and intubation, fungemia due to *C. albicans* | Fever, severe bronchopneumonia | Blood inside the CVCs | 10 Y/F |  | AMB, FLZ, VCZ, FLC, CAS | *C. metapsilosis* | Kuwait 2016 |
| (Alencar et al, 2017) | Treated |  | FLZ | CRF, DM, CVC | DM, CRF, endocarditis | Blood | 33 D/- |  |  | *C. orthopsilosis* | Brazil 2017 |
| (Alencar et al, 2017) | Treated |  | FLZ | PB, CVC |  | Blood | <1 D/- |  |  | *C. orthopsilosis* | Brazil 2017 |
| (Charsizadeh et al, 2018) | Died | - | AMB, FLZ | Abdominal surgery, CVC, TPN, TI | Prematurity, respiratory disorder | Blood | 18 D/F | - | - | *C. orthopsilosis* | Iran 2018 |
| (Charsizadeh et al, 2018) | Treated | - | AMB, FLZ | Surgery, CVC, TPN, TI | Prematurity, neurological and respiratory disorder | Blood | 28 D/M | - | - | *C. orthopsilosis* | Iran 2018 |
| (Charsizadeh et al, 2018) | Treated | - | AMB | CVC, steroid therapy, TI | B cell leukemia | Blood | 3 Y/F | - | - | *C. orthopsilosis* | Iran 2018 |
| (Charsizadeh et al, 2018) | Died | - | AMB | CVC, TPN, TI | Metabolic and gastrointestinal disorder | Blood | 12 Y/F | - | - | *C. orthopsilosis* | Iran 2018 |

D: day, Mo: month, Y: year, F: female, M: male, DALK: deep anterior lamellar keratoplasty due to keratoconus, CRF: chronic renal failure, DM: diabetes mellitus, PB: preterm birth, CVC: central venous catheter, TI: tracheal intubation; TPN: total parenteral nutrition, FLZ: fluconazole, ITZ: itraconazole, VCZ: voriconazole, AMB: amphotericin B, PSZ: posaconazole, FLC: flucytosine (5-FC), KTZ: ketoconazole, CAS: caspofungin, SDD: susceptible dose-dependent

**References:**

Alencar, D. de S. O. de, Tsujisaki, R. A. de S., Spositto, F. L. E., Nunes, M. de O., Almeida, A. A. de, Martins, M. D. A., et al. (2017). Candidaemia due to *Candida parapsilosis* species complex at a hospital in Brazil: Clinical characteristics and antifungal susceptibility profile. *Revista iberoamericana de micologia* 34, 106–108. doi:10.1016/j.riam.2016.06.008.

Arezoo Charsizadeh , Hossein Mirhendi , Bahram Nikmanesh, Hamid Eshaghi, Maryam Rahmani , Armin Farhang, H. B. and K. M. (2018). Candidemia in Children Caused by Uncommon Species of *Candida*. *Archives of Pediatric Infectious Diseases* 6, e11895.

Asadzadeh, M., Ahmad, S., Al-Sweih, N., Gulati, R. R., and Khan, Z. (2016). First isolation of *Candida metapsilosis* in Kuwait, an emerging global opportunistic pathogen. *Journal de mycologie medicale* 26, 46–50. doi:10.1016/j.mycmed.2015.11.001.

Choi HJ, Shin JH, Park KH, Shin MG, Suh SP, R. D. (2010). A Fatal Case of *Candida orthopsilosis* Fungemia. *Korean Joournal of Clinical Microbiology* 13, 140–143.

Heslop, O. D., De Ceulaer, K., Rainford, L., and M Nicholson, A. (2015). A case of *Candida orthopsilosis* associated septic arthritis in a patient with Systemic Lupus Erythematosus (SLE). *Medical mycology case reports* 7, 1–3. doi:10.1016/j.mmcr.2014.11.001.

Oliveira, V. K. P., Paula, C. R., Colombo, A. L., Merseguel, K. B., Nishikaku, A. S., Moreira, D., et al. (2014). Candidemia and death by *Candida orthopsilosis* and *Candida metapsilosis* in neonates and children. *Pediatrics and neonatology* 55, 75–76. doi:10.1016/j.pedneo.2013.07.006.

Wessel, J. M., Bachmann, B. O., Meiller, R., and Kruse, F. E. (2013). Fungal interface keratitis by *Candida orthopsilosis* following deep anterior lamellar keratoplasty. *BMJ case reports* 2013. doi:10.1136/bcr-2012-008361.
